# Exploring Medical Students’ Representations of Future Specialties and Parenthood: Protocol for a Scoping Review

**DOI:** 10.2196/78133

**Published:** 2026-01-20

**Authors:** Sylvie Arnoux, Mia Gisselbaek, Georges Louis Savoldelli, Nadia Masood Bajwa

**Affiliations:** 1 Unit of Development and Research in Medical Education, Faculty of Medicine, University of Geneva, 1 rue Michel Servet, Geneva, 1211, Switzerland, 41 22 379 59 35; 2 Department of Anaesthesiology, Pharmacology, Intensive Care and Emergency Medicine, Division of Anaesthesiology, University Hospital of Geneva, Geneva, Switzerland; 3 Department of Women, Children, and Adolescents, Division of General Pediatrics, University Hospital of Geneva, Geneva, Switzerland

**Keywords:** medical student, parenthood, maternity, specialty choice, representations, career choice, scoping review, family planning

## Abstract

**Background:**

Several factors come into consideration when medical students choose their future specialty. Among these factors, the desire to start a family and planning the best timing for pregnancy may interfere with career advancement in certain specialties.

**Objective:**

To the best of our knowledge, this is the first scoping review aimed at understanding medical students’ career choice and parental expectations without restriction of the specialty chosen. This protocol describes a scoping review aiming to understand how representations regarding specialties and parenthood influence medical students’ career choice.

This protocol describes a scoping review aiming to understand how representations regarding specialties and parenthood influence medical students’ career choice.

**Methods:**

We will search PubMed, Embase, Web of Science, ERIC, and PsycInfo for literature. Additionally, the reference lists of included articles will be screened for further inclusion. Rayyan and Endnote will be used to organize data screening and extraction. The database selection will allow us to extract and analyze data from various disciplines. This diversity will increase our understanding of medical students’ career and personal life decisions. This protocol and the upcoming scoping review have been designed following the PRISMA-ScR (Preferred Reporting Items for Systematic Reviews and Meta-Analyses extension for Scoping Reviews) guidelines to ensure the quality of the searching process, the data screening, and the data extraction.

**Results:**

This study will conduct a thematic synthesis of how the concepts of representations and perceptions of parenthood are used by medical students in the selected literature, comparing them to theoretical frameworks to clarify their meanings. We also plan to identify key themes related to parenthood and medical specialty choice when planning a career. As of December 2025, we proceeded to data screening. We anticipate publishing our results in the second quarter of 2026.

**Conclusions:**

This scoping review aims to better understand medical students’ representations of medical specialties and parenthood, and how these perceptions influence their specialty preferences and career choices. By mapping existing evidence across various disciplines, the review will identify research gaps and provide a foundation for future studies. The findings will offer valuable insights into the challenges of balancing career aspirations and family life, particularly in the context of physician shortages and the growing feminization of the medical profession.

## Introduction

As medical students complete their studies and start their residency, several factors come into consideration when choosing a career path. For both men and women, initial career intentions may waver as personal life factors are taken into consideration [1-4].

As they complete their initial training, female students often anticipate that their work and their personal life may conflict [1]. When planning their future career, female medical students may take into consideration their desire to become a parent, the best time to have a child, parental obligations, and the tensions created by these responsibilities [5,6]. These reflections lead medical students to delay pregnancy despite the increased risk of infertility and pregnancy complications [7]. Furthermore, becoming a mother may expose women physicians to discrimination, lack of career opportunities, and limited support from the institution [6,8,9]. Working part-time to take care of children may diminish career opportunities or prolong residency training [10,11]. Social norms, medical culture, and institutional structure and policies are possible sources of this discrimination [8,12,13].

Supervisors and more senior colleagues encountered in the clinical setting are role models for medical students. Role models help students project themselves in a desired specialty [5,14]. Experiences during clerkships and residency allow medical students to synthetize what they see and hear to build their representations [14-16]. However, a lack of role models, especially female ones, can affect how female medical students perceive women’s position within the specialty and its environment [5,17,18]. The lack of female mentors can also affect learning and students’ motivation [19]. Furthermore, students might encounter more fathers than mothers among their colleagues, especially in specialties where women are underrepresented [20].

Colleagues and supervisors’ opinions regarding a specialty and the commitment it demands, their banter, and their comments about their past personal experiences influence students’ projections about their future career [5,21-23]. Among those remarks, comments on how difficult it can be to reconcile maternity and career advancement affect the construction of career representations and motivation to pursue a specific specialty [2,17,24]. Such negative attitudes from colleagues contribute to reinforcing negative stereotypes, which in turn generate stereotype threats [25]. Female physicians might embody these negative stereotypes and unconsciously identify themselves with those stereotypes and expose themselves to self-sabotaging [25]. These factors underline the importance of conducting a scoping review on medical student career choices and the influence of parenthood aspirations.

As medical students progress through their training, they face the challenge of envisioning their future as doctors while balancing personal aspirations and life goals. When choosing a specialty, they must take into consideration several factors, such as family planning and the requirements of residency. Starting a career while becoming a parent can be very challenging.

Although there are a number of studies addressing career choice in relation to physician shortages, little is known about the aspirations and representations of medical students regarding their future career as a doctor and their private life. A systematic review has been conducted on experiences and perspectives of women who are already doctors and mothers and focuses on how the family life of trained doctors and their career conflict [6]. Another review has focused on surgery and how the working environment may generate specific challenges, such as long hours working and prolonged periods of standing, and higher rates of pregnancy complications [26]. The use of the terms representations or perception often lacks definition or operationalization in the literature. Drawing on social representation theory will help better define and conceptualize these terms [27-29].

To the best of our knowledge, no comprehensive review has synthetized the intersection of medical students’ career perspectives, family planning, and specialty choice. Understanding these interconnected factors is crucial as they influence workforce planning, the well-being of future health care professionals, and the alignment of personal and professional aspirations. The findings may be useful to decrease the attrition of junior doctors when entering practice.

A scoping review allows for a comprehensive exploration of the existing literature, identifying gaps and patterns related to how parental responsibilities and representations influence career trajectories in medicine. Such a review can provide valuable insights into the societal, institutional, and cultural narratives that shape medical students’ decisions.

We intend to synthesize existing literature on the representations and aspirations of family planning and career choice of medical students across medical specialties to answer the following research question: How do these representations influence medical students’ career choice and desire to become parents?

## Methods

### Overview

Since we are interested in understanding how medical students project themselves into their career and parenthood and we aim to explore evidence on the topic, we will conduct a scoping review. The review will adhere to the PRISMA-ScR (Preferred Reporting Items for Systematic Reviews and Meta-Analyses extension for Scoping Reviews) principles [30,31].

Preliminary searches occurred between August and November 2024 to establish the search strategy. This iterative process helped refine the choice of keywords and relevant databases.

The present protocol may undergo further revision as designing and writing a scoping review is an iterative process. Deviations from the protocol will be explained in the future manuscript to ensure transparency.

### Information Sources

The following databases were searched: PubMed, Embase, Web of Science, ERIC, and PsycInfo. Additionally, reference lists of included articles and Google Scholar results have been screened for further inclusion. We focused on peer-reviewed data to map evidence broadly. Excluding gray literature aligns with this exploratory purpose, emphasizing breadth and clarity over exhaustive comprehensiveness. The data collection took place in February and March 2025. The search strategy was elaborated with the help of a librarian specializing in systematic searches. An example of the search strategy is available in Multimedia Appendix 1. The key concepts used to develop the search strategy are outlined in Table 1. The specific thesauri of each database were consulted to identify the most appropriate terminology. A selection of relevant articles was established to test the accuracy of the search strategy.

**Table 1.** Key concepts.

Subject		Becoming a parent
Med* student*	Specialt*	Parent*
Med* school student*	Career choice	Motherhood
Medical education	Occupational choice*	Fatherhood
	Occupational aspiration*	Family planning
	Job selection*	Life change*
	Career aspiration*	Having children
	Career goal*	Childbearing
	Vocational aspiration*	Maternity
	Goal orientation*	Pregnancy

### Inclusion Criteria

To be included, studies must report on medical students and their career choice, family planning, and representations about having a family while starting a career (Textbox 1). As our goal is to map existing evidence regarding our subject, inclusion criteria do not include geographic area, a specific specialty, or a language. This choice reflects the diversity of existing situations and representations regarding the interaction between parenthood, being a doctor, and medical specialties. As our work is focused on medical students, we aim to explore recent and representative problematics. The review includes publications from 2000 to the present. Studies are excluded if they focus only on medical residents, nursing students, or physicians; on work-life balance only; or on policies.

Textbox 1. **Inclusion and exclusion criteria.**

**Inclusion criteria**

- Focusing on medical students
- Address parenthood aspiration/desire/representation
- Any geographical area
- Any language
- Published from 2000

**Exclusion criteria**

- Focusing on residents, physicians, or nursing students
- Medical students’ parents
- Focus on policies
- Published before 2000

Representations here are defined as values, beliefs, and experiences that interact to help individuals make sense of their experiences [27]. These factors can be shared and built collectively, but individuals interpret them differently. Furthermore, these representations are embedded in a sociocultural and historical context that encompasses various ways of sharing and transmitting representations. Itkonsen et al [29] have previously used social representations to understand the factors related to career choice and planning among university students. Expectations and stereotypes of the role of a doctor and daily life in a specific specialty shape the representation of their career before starting clinical rotations. As medical students are confronted with the reality of the clinical ward and private practice, their experiences enrich their representations. Sharing experiences with their peers also contributes to building their representations. A similar process occurs when students project themselves in their future career and personal lives.

The research team has an academic understanding of French, English, Italian, and Spanish. To ensure best understanding, titles and abstracts of studies published in another language were translated into English using DeepL [32]. The full article will be translated if it is included after the screening process. Both the original and translated version will be used for data extraction. The translated articles will be assigned to researchers based on their comprehension of the original language. Full documentation of the process will be detailed using the PRISMA-ScR chart [31].

A vocabulary list has been established for each key concept and adjusted to the selected database.

### Data Screening

The search results were exported in Rayyan [33], a program designed to conduct systematic reviews, identify and remove duplicates, and provide a comprehensive list of all criteria and associated questions. Preliminary research indicated that only a relatively small number of articles were likely to be found from the research equations, enabling all authors (SA, NMB, MG, and GLS) to collaboratively proceed with the title and abstract screening to ensure consistency. The previously defined eligibility criteria were applied to determine whether each article should be included. Regular meetings were held to discuss the inclusion or exclusion of the articles for which no consensus has emerged during the iterative process.

The selected articles were exported to EndNote 21 (Clarivate) [34] and underwent a full-text screening. Any studies recommended for exclusion at this stage were reviewed by SA, NMB, and MG to ensure that exclusion criteria were met. Reference lists of each selected article will be screened to ensure that relevant references are included. Adjustments to the inclusion or exclusion criteria were discussed and agreed upon among the authors throughout the search and screening process.

### Data Extraction

Articles meeting the inclusion criteria after the first screening will be listed in an Excel file (Microsoft Corp) providing key elements regarding their content. First, studies will be sorted by characteristics, including country of origin, study design, and data collected, if any. Then, studies will be thematically sorted. Themes of interest involve the conciliation of career choice and desires for parenthood, representations regarding medical specialties and their openness to parenthood, specific challenges regarding either motherhood or fatherhood, or representations about medical specialties and parenthood in general. This list has been established based on the literature cited in the Introduction and findings gathered in interviews during an ongoing project (S Arnoux et al, MSc, unpublished, 2024 data).

We will conduct a critical appraisal of individual sources of evidence to assess their relevance, reliability, validity, and applicability. The Joanna Briggs Institute provides a quality assessment tool specific to each study design type included in the studies [35]. This step will increase the quality of the scoping review and facilitate data collection and analysis.

SA and NMB will perform the data extraction independently. GLS and MG will independently check 20% of the extracted data to ensure that extraction criteria are met. Two meetings will be held at the beginning and the end of the extraction process to define the theme list and discuss the completed chart to reach consensus regarding the extracted data. If necessary, the themes list will be discussed and adjusted during the extraction process to include emerging themes. A final version of the chart will be provided in the scoping review.

### Ethical Considerations

Ethics approval will not be necessary as this project does not fall under the Swiss law for human research (LRH) [36]. The scoping review will be published in a scientific journal and its results will be presented at conferences. This scoping review is part of a bigger project focusing on Swiss medical students’ career choices. This project aims to understand and describe the factors influencing the career choices of medical students to inform stakeholders and address the workforce shortage.

## Results

Representations and perceptions are often used in literature but rarely conceptualized. We intend to conduct a thematic synthesis of the terms used in the selected studies and compare this with the theoretical literature to provide a synthesis of what is understood when speaking of representations. Furthermore, we will describe the main themes associated with parenthood and medical specialty choice.

**Figure 1.**
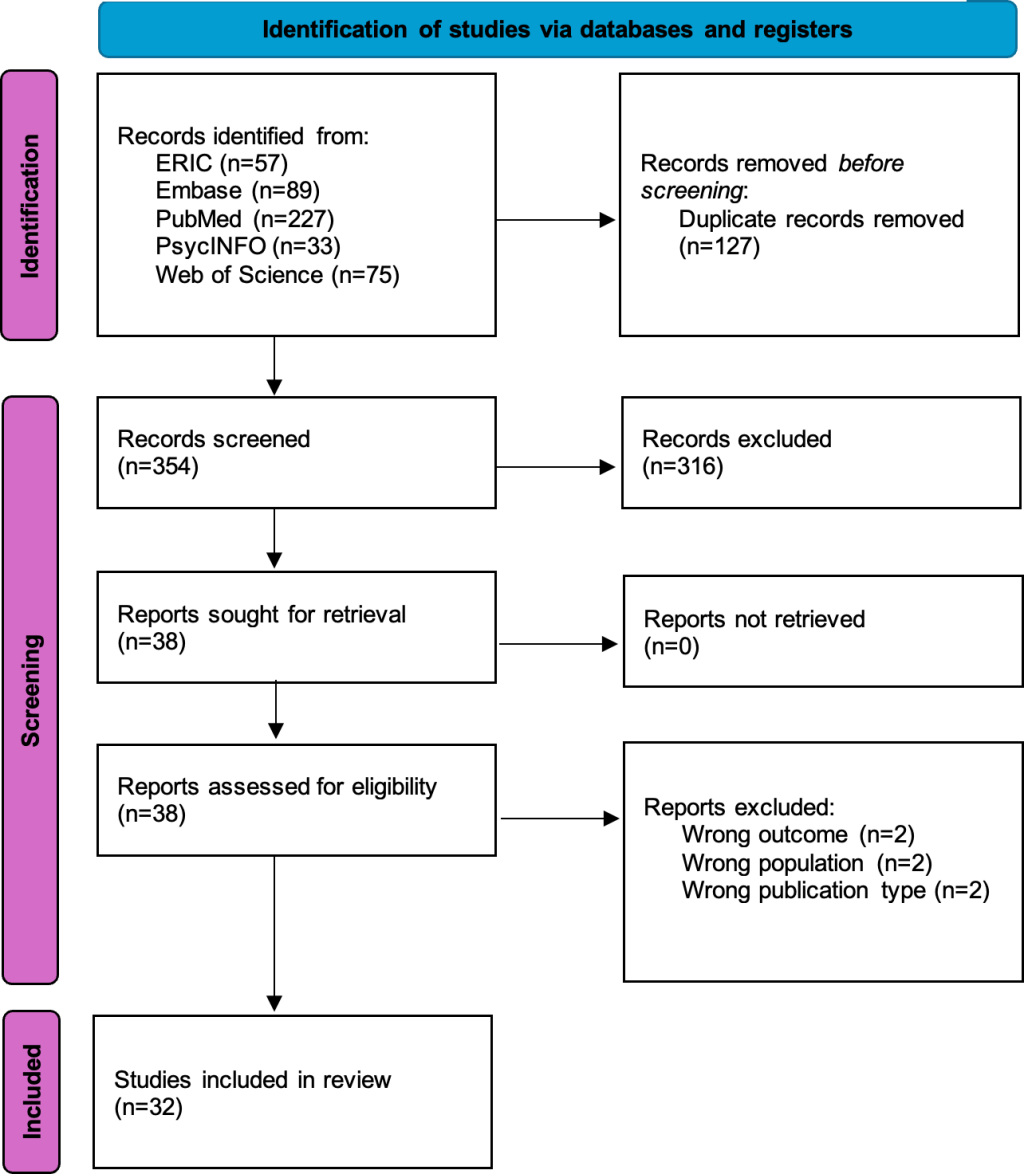
PRISMA (Preferred Reporting Items for Systematic Reviews and Meta-Analyses) flow chart for scoping reviews [37].

A total of 32 articles have been identified (Figure 1). As of December 2025, data extraction has started. We expect to publish the results in the second quarter of 2026. This project obtained a grant from the Swiss National Science Foundation in October 2022.

## Discussion

This scoping review aims to provide a better understanding of medical students’ representations of medical specialties and parenthood. The main objective is to improve our comprehension of the impact of their representations on their specialty preferences and career choices.

Previous studies have focused on specific specialties (such as surgery) and how physicians, residents, or students make decisions regarding their career or their family planning. This scoping review will focus on medical students in various disciplines and the influence of representations on their plans regarding their future career and personal life.

This scoping review will identify and map the existing evidence regarding medical students’ representations regarding the interaction between parenthood and medical specialty. Synthesizing the existing evidence will shed light on the gaps and the potential for further research. In the context of physician shortages and feminization of the medical profession, the results of the scoping review will provide valuable insight to address the challenges faced by physicians to reconcile their career and family planning.

## Supplementary material

10.2196/78133 Multimedia Appendix 1 Example of search strategy: PubMed.

10.2196/78133 Checklist 1 PRISMA checklist.
