# Supplementary material for: Exploring Medical Students’ Representations of Future Specialties and Parenthood: Protocol for a Scoping Review
Source: JMIR Res Protoc. 2026 Jan 20;15:e78133. doi: 10.2196/78133 (PMC12818495; doi:10.2196/78133)
Supplement: Multimedia Appendix 1 [file resprot-v15-e78133-s001.docx]

Multimedia Appendix 1

Example of search strategy : PubMed

("education, medical"[MeSH Terms] OR "education, medical, graduate"[MeSH Terms] OR "students, medical"[MeSH Terms] OR "Medical Student"[Title/Abstract] OR "Medical Students"[Title/Abstract] OR "med student"[Title/Abstract] OR "medical school student"[Title/Abstract:~1] OR "Medical education"[Title/Abstract]) AND ("Parents"[MeSH Terms] OR "Parenthood"[Title/Abstract] OR "Parent"[Title/Abstract] OR "Parents"[Title/Abstract] OR "Parental Age"[Title/Abstract] OR "Parental Ages"[Title/Abstract] OR "Parenting"[Title/Abstract] OR "Motherhood"[Title/Abstract] OR "Fatherhood"[Title/Abstract] OR "Parental leave"[Title/Abstract] OR "Parenthood Status"[Title/Abstract] OR "Parentage"[Title/Abstract] OR "Family Planning"[Title/Abstract] OR "childbearing"[Title/Abstract] OR "having children"[Title/Abstract]) AND ("Career Choice"[MeSH Terms] OR "Career Choice"[Title/Abstract] OR "Medical specialty"[Title/Abstract] OR "Medical specialties"[Title/Abstract] OR "Specialty choice"[Title/Abstract] OR "Specialty choices"[Title/Abstract])
